# Beyond the Bones: A Case Report of Extraosseous Ewing Sarcoma

**DOI:** 10.7759/cureus.47961

**Published:** 2023-10-30

**Authors:** Ana Margarida Simões, Joana Sequeira-Mendes, Ana Isabel Santos

**Affiliations:** 1 Family Medicine, Unidade de Saúde Familiar A Ribeirinha, Unidade Local de Saúde Guarda, Guarda, PRT

**Keywords:** ewing, sarcoma soft tissue, abdominal pain, ewing sarcoma (es), liver mass, multidisciplinary care approach

## Abstract

Ewing sarcoma (ES) is primarily recognized as a primary bone tumor; however, its extraosseous variant is exceptionally rare and presents unique clinical challenges. In this article, we report the case of a 22-year-old male who initially presented with abdominal swelling. Diagnostic tests included abdominal imaging and a CT scan, revealing a solid liver mass. A thorough evaluation confirmed it to be an extraosseous ES, supported by liver biopsy and immunohistochemistry demonstrating positive expression for AE1/AE3 and CD-99, along with genetic analysis revealing a rearrangement of the *EWSR1 *gene (translocation 22q12). The patient’s treatment involved a multimodal approach, including perioperative chemotherapy, surgery, and postoperative chemotherapy, following which the patient remained in complete remission after 24 months. This case emphasizes the importance of considering rare malignancies such as ES in differential diagnoses for young patients with liver masses. It also accentuates the pivotal role of family physicians in early detection and holistic patient care, underscoring the need for comprehensive investigations when encountering persistent symptoms.

## Introduction

Ewing sarcoma family of tumors (ESFT) represents a rare and intricate group of malignancies originating from mesenchymal tissues [1]. These tumors cover a range of neuroectodermal differentiation and share distinct morphological and cytogenetic attributes. Within this family, the ESFT spectrum comprises several entities, including extraosseous Ewing sarcoma (ES), primitive neuroectodermal tumor, Askin’s tumor, and atypical ES [1,2]. Although ESFT predominantly affects children and young adults, typically involving the bones, it is important to highlight that these tumors can also manifest in soft tissues, without any bone involvement. Extraosseous ES is most frequently located within deep soft tissues, the retroperitoneum, and the chest wall, occasionally appearing in visceral organs, such as the liver [1].

The exceptional rarity of extraosseous ES adds a layer of complexity to its clinical presentation and management. ESFT, known for its inherent aggressiveness and tendency to recur and metastasize, often involving sites such as the lungs, bone marrow, brain, and lymph nodes, underlines the critical need for in-depth exploration of these unique tumor manifestations [3,4]. As the understanding of ESFT and its extraosseous variants progresses, this case contributes to improving our understanding of these uncommon malignancies and developing strategies for their optimal management.

## Case presentation

A 22-year-old man with no significant personal or family history (including an absent family history of cancer) came to the primary care consultation due to painful abdominal swelling and an unintentional 10 kg weight loss that had developed over the last three months. The patient reported having gone to the emergency department for two episodes of nausea, vomiting, diarrhea, and abdominal pain, which were diagnosed and treated as acute gastroenteritis two weeks before. Upon physical examination, a soft and depressible abdomen with tenderness in the right hypochondrium was observed. Deeper palpation revealed an enlarged liver with regular borders extended approximately 5 cm below the costal margin. Laboratory findings (Table 1) showed a slight elevation in gamma-glutamyl transferase at 108 U/L and alanine aminotransferase at 66 U/L. Serum tumor markers, including alpha‐fetoprotein and carcinoembryonic antigen, were negative and carbohydrate antigen 19-9 showed a slight increase of 48 U/mL. Serologies for hepatitis B and C virus were negative.

**Table 1:** Laboratory test results with normal ranges (altered values are indicated in bold).

Laboratory tests	Patient’s values	Normal range
Hemoglobin (g/dL)	14.5	13.0–17.0
Leukocytes (µL)	7.7 × 10^3^	4.0–10.0 × 10^3^
Platelets (µL)	322 ×10^3^	150–400 ×10^3^
Creatinine (mg/dL)	1.64	0.70–1.30
Aspartate aminotransferase (U/L)	32	15–37
Alanine aminotransferase (U/L)	66	16–63
Gamma-glutamyl transferase (U/L)	108	15–85
Total bilirubin (mg/dL)	0.38	0.20–1.00
Direct bilirubin (mg/dL)	0.11	0.00–0.20
Hepatitis B surface antigen	<0.10 (negative)	Negative <1.00 Positive ≥1.00
Hepatitis C antibody	0.33 (negative)	Negative <0.80 Positive ≥1.00
Alpha-fetoprotein (ng/mL)	1.91	<7.00
Carcinoembryonic antigen (ng/mL)	2.90	0.0-4.5
Carbohydrate antigen 19-9 (U/mL)	47.9	<39.0
Antibodies against smooth muscle	Negative	-
Anti-mitochondrial antibodies	Negative	-
Anti-liver-kidney microsomal antibodies	Negative	-
Anti-gliadin IgA antibodies (U/mL)	3.1	Negative <7 Positive >10
Anti-gliadin IgG antibodies (U/mL)	2.2	Negative <7 Positive >10
Transglutaminase IgG (U/mL)	0.9	Negative <7 Positive >10
Transglutaminase IgA (U/mL)	1.2	Negative <7 Positive >10

**Figure 1:**
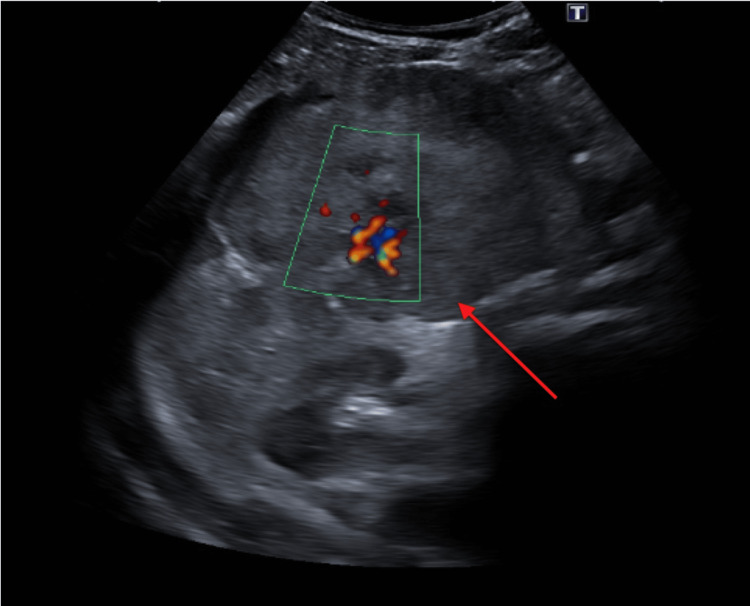
Abdominal ultrasonography showing a solid nodular formation measuring 113 x 94 mm with a color Doppler signal involving the left lobe and part of the right hepatic lobe.

**Figure 2:**
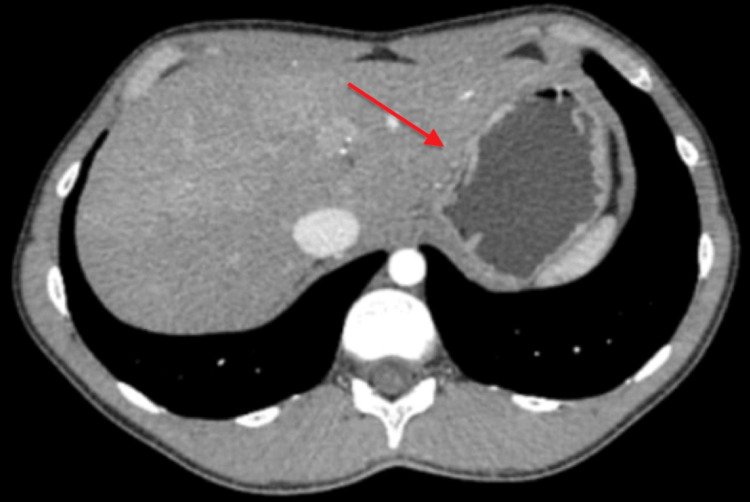
Abdominal contrast-enhanced CT showing a large, heterogeneous, lobulated, well-defined mass, apparently originating from the IV B segment, the V segment, and a portion of the VI segment of the liver, as indicated by the red arrow.

Abdominal ultrasound (Figure 1) and abdominal contrast-enhanced CT (Figure 2) revealed a solid hepatic mass measuring 130 x 100 mm in segments IV B, V, and part of segment VI.

The patient was then referred to the hospital, where a percutaneous biopsy of the liver mass was performed. The biopsy revealed fragments of malignant neoplasm, densely cellular, composed of small cells with a rounded, vesicular, or hyperchromatic nucleus. The cells were arranged in a towel-like pattern, mixed with a scarce inflammatory infiltrate within a dense, fibrous streak. Immunohistochemical analysis showed positive expression for AE1/AE3 and CD-99. In addition, a genetic analysis of the sample revealed a rearrangement of the *EWSR1 *gene (translocation 22q12). These findings supported the diagnosis of ES. The case was presented to a multidisciplinary team to discuss the optimal therapeutic approach. Chemotherapy was initiated before surgery to reduce the size of the lesion consisting of six cycles of vincristine, doxorubicin, and cyclophosphamide followed by mesna (sodium 2-mercaptoethane sulfonate).

After the third cycle, this scheme had to be alternated with ifosfamide and etoposide followed by mesna due to complications and toxicity (severe pancytopenia, bacteremia, and pseudomembranous enterocolitis). After completing six cycles of chemotherapy, there was a significant reduction in the lesion, measuring approximately 5 cm in size. A fluorodeoxyglucose (FDG) positron emission tomography/computed tomography (PET/CT) was then performed to confirm the complete metabolic response. The patient underwent laparoscopic resection of the hepatic segments IV B/V of the liver, which contained the tumor, along with laparoscopic cholecystectomy due to apparent sarcoma invasion.

Post-surgery, the anatomopathological analysis revealed a neoplastic mass measuring 4.4 cm with a transmural invasion of the gallbladder wall. Histological examination revealed the presence of neoplastic cells in the surgical margins. Due to the patient’s history and preferences, the decision was made to opt for adjuvant radiotherapy without chemotherapy.

The patient is currently under clinical surveillance and has remained in complete remission for 24 months.

## Discussion

ES, particularly its extraosseous variant, represents a diagnostic and therapeutic challenge due to its rarity and often ambiguous initial clinical presentation [5].

Although the primary bone form of ES is well-documented, extraosseous ES, as seen in our patient, can mimic other benign or malignant conditions and often leads to a delay in definitive diagnosis [3]. ES frequently affects adolescents and young adults, making it imperative for clinicians to have a high suspicion when encountering atypical soft tissue masses in this population [6]. As seen in our patient, abdominal pain or discomfort is a common initial symptom, yet such non-specific complaints may divert the initial workup toward more common conditions, such as hepatic infections or benign liver lesions, thereby delaying the correct diagnosis [1].

The role of imaging in diagnosing ES is crucial. Although imaging findings are often non-specific, certain patterns emerge based on the tumor’s location [7]. Extraosseous ESs are usually large, bulky soft-tissue masses that may display heterogeneity due to necrosis or hemorrhage. A typical presentation involves a large tumor with central necrosis that does not cross the midline. MRI is often the preferred modality for assessing the primary tumor and local staging [8]. CT and fluorine 18 FDG PET/CT are highly sensitive in detecting distant metastases, especially in the lungs and lymph nodes [9]. In the diagnostic process, ultrasonography provides an initial assessment; however, CT and MRI offer more comprehensive insights into the lesion’s characteristics and extent, as well as its relationship with neighboring structures. This helps guide biopsy and treatment planning, as was the case in our patient [8,9].

Small round blue cells, a feature found in ESFT, are not unique to this tumor and can also be observed in other malignancies such as lymphoma and rhabdomyosarcoma [8]. The diagnosis of ESFT hinges on distinct immunohistochemical staining patterns and cytogenetic or molecular tests [10]. Notably, ESFT is characterized by strong membranous CD99 positivity, a cell surface glycoprotein that, although prevalent in ESFT, is not exclusive to it. Molecular confirmation techniques, such as fluorescence in-situ hybridization or next-generation sequencing, have become commonplace, mainly due to the presence of *EWSR1 *rearrangements in approximately 90% of ES cases [11]. This translocation joins the *EWSR1 *gene on chromosome 22 with the *FLI1 *gene on chromosome 11 (t(11;22)(q24;q12)), creating the EWSR1-FLI1 fusion product, known for its oncoprotein functions [8,10,11]. The presence of a positive CD99 in IHC, combined with the *EWSR1 *gene rearrangement, as seen in our patient, confirms the diagnosis of ES and distinguishes it from its mimickers.

Given the infrequent occurrence of ESFT, a dedicated staging system has not been developed. Instead, it is recommended to use the broader American Joint Committee on Cancer staging system designed for all soft-tissue sarcomas [8,9]. The rarity of liver ESFT cases makes it challenging to establish standard treatment guidelines, and more data are needed to determine the optimal management approach. Surgery alone is generally considered insufficient, primarily due to the tumor’s size and the potential for metastases to develop [1]. Therefore, it is recommended to add chemotherapy and/or radiation therapy, as was the case in our patient, to enhance the likelihood of achieving prolonged remission and improved survival rates [1,8,9]. Neoadjuvant chemotherapy is suggested to target presumed micrometastases and reduce the size of the primary tumor [9]. While ESFT is known to be responsive to radiation therapy, its use has been reduced due to increased complications, particularly in skeletally immature patients [8]. Presently, it is primarily reserved for cases where achieving local control through surgery is not feasible, for instances with inadequate surgical margins, or in patients with a limited response to chemotherapy [8,9]. In North America, the standard adjuvant chemotherapy regimen typically includes vincristine, doxorubicin, and cyclophosphamide, with alternating cycles of ifosfamide and etoposide [8].

Follow-up and surveillance are critical, as the risk of local recurrence or metastatic spread persists. According to published guidelines, regular follow-up after therapy of primary ES includes CT of the chest (to detect lung metastases in asymptomatic patients) every three months for the first two years and every six months for the first five years. After the five-year surveillance, a CT of the chest is recommended once or twice per year up to 10 years after the completion of treatment. Long-term surveillance beyond 10 years should be performed based on clinical indications [9,11].

## Conclusions

ES is a rare type of small round-cell tumor that lacks specific clinical or radiological features. Diagnosis requires histopathological, immunohistochemical, and cytogenetic examinations. Given the rarity of extraosseous ES, further studies and research are required to enhance the understanding of this condition and develop more effective treatment strategies. This case presentation significantly contributes to the medical literature, emphasizing the importance of maintaining vigilance toward uncommon symptoms, especially among young patients. The family physician plays a crucial role as the initial link in the healthcare chain, highlighting the significance of ongoing education and interdisciplinary collaboration in diagnosing and treating challenging medical conditions such as extraosseous ES. Early recognition remains a determining factor that can have a substantial impact on the prognosis of these patients.

## References

[1] Hedfi M, Ben Ismail I, Zenaidi H, Bouslama S, Zoghlami A (2022). Primary Ewing sarcoma of the liver: diagnosis, management, and prognosis: a case report and literature review. Clin Case Rep.

[2] Ozaki Y, Miura Y, Koganemaru S (2015). Ewing sarcoma of the liver with multilocular cystic mass formation: a case report. BMC Cancer.

[3] Cambruzzi E, Guerra EE, Hilgert HC (2011). Primitive neuroectodermal tumor of the liver: a case report. Case Rep Med.

[4] Mani S, Dutta D, De BK (2010). Primitive neuroectodermal tumor of the liver: a case report. Jpn J Clin Oncol.

[5] Ousadden A, Mazaz K, Amraoui A, Kettani F, Chefchaouni MC, Ait Taleb K (2005). [Primary hepatic localization of the PPNET (primitive peripheral neuroectodermal tumors). Case report]. Ann Chir.

[6] Ferrari A, Bleyer A, Patel S, Chiaravalli S, Gasparini P, Casanova M (2018). The challenge of the management of adolescents and young adults with soft tissue sarcomas. Pediatr Blood Cancer.

[7] Lu T, Yang W, Liu X, Yang X, Yang C, Di W (2022). Imaging findings of hepatic Ewing's sarcoma on computed tomography and gadobenate dimeglumine-enhanced magnetic resonance imaging: a case report and literature review. J Clin Transl Hepatol.

[8] Wright A, Desai M, Bolan CW (2022). Extraskeletal Ewing sarcoma from head to toe: multimodality imaging review. Radiographics.

[9] Casali PG, Abecassis N, Aro HT (2018). Soft tissue and visceral sarcomas: ESMO-EURACAN Clinical Practice Guidelines for diagnosis, treatment and follow-up. Ann Oncol.

[10] Pinto A, Dickman P, Parham D (2011). Pathobiologic markers of the ewing sarcoma family of tumors: state of the art and prediction of behaviour. Sarcoma.

[11] Digklia A, Dolcan A, Kucharczyk MA, Jones RL, Napolitano A (2023). Optimal delivery of follow-up care following treatment for adults treated for Ewing sarcoma. Cancer Manag Res.

